# Early growth, dominance acquisition and lifetime reproductive success in male and female cooperative meerkats

**DOI:** 10.1002/ece3.820

**Published:** 2013-10-09

**Authors:** Sinead English, Elise Huchard, Johanna F Nielsen, Tim H Clutton-Brock

**Affiliations:** 1 Large Animal Research Group, Department of Zoology, University of CambridgeCambridge, CB2 3EJ, UK; 2 School of Biological Sciences, Institute of Evolutionary Biology, University of EdinburghWest Mains Road, Edinburgh, EH9 3JT, UK; 3 Institute of Zoology, Zoological Society of LondonRegent's Park, London, NW1 4RY, UK; 4 Mammal Research Institute, Department of Zoology and Entomology, University of PretoriaPretoria 0002, South Africa

**Keywords:** Cooperative breeders, early development, female competition, reproductive success

## Abstract

In polygynous species, variance in reproductive success is higher in males than females. There is consequently stronger selection for competitive traits in males and early growth can have a greater influence on later fitness in males than in females. As yet, little is known about sex differences in the effect of early growth on subsequent breeding success in species where variance in reproductive success is higher in females than males, and competitive traits are under stronger selection in females. Greater variance in reproductive success has been documented in several singular cooperative breeders. Here, we investigated consequences of early growth for later reproductive success in wild meerkats. We found that, despite the absence of dimorphism, females who exhibited faster growth until nutritional independence were more likely to become dominant, whereas early growth did not affect dominance acquisition in males. Among those individuals who attained dominance, there was no further influence of early growth on dominance tenure or lifetime reproductive success in males or females. These findings suggest that early growth effects on competitive abilities and fitness may reflect the intensity of intrasexual competition even in sexually monomorphic species.

## Introduction

In polygynous species, reproductive competition is more intense among males than females (Clutton-Brock 1988), and as such males may experience stronger selection for competitive traits (Emlen and Oring 1977). Early development has lasting effects on adult phenotype and associated fitness across a range of taxa (Lindström 1999; Lummaa and Clutton-Brock 2002; Monaghan 2008). In species with stronger selection for competitive traits in males compared to females, early growth conditions can have sex-specific fitness effects (e.g., red deer, *Cervus elaphus*, Kruuk et al. 1999; bighorn sheep, *Ovis canadensis*, Festa-Bianchet 2000, LeBlanc et al. 2001). Such variation is often associated with striking sexual size dimorphism (Badyaev 2002), although this is not always the case (e.g., humans, Kuzawa et al. 2010).

Cooperative breeding vertebrates, where several adults forgo independent reproduction to assist raising the young of others, offer an interesting contrast to the picture above. Competition among females over access to resources necessary for reproduction is often high in these species, which can lead to stronger selection for competitive traits in females (Hauber and Lacey 2005; Clutton-Brock 2009). In meerkats, for example, variance in reproductive success is higher among females than among males (Clutton-Brock et al. 2006), and size-associated traits at adulthood have greater fitness consequences for females (Clutton-Brock et al. 2006). Unlike polygynous species, however, extreme sexual size dimorphism does not result, potentially as a consequence of limits to fecundity in females (Clutton-Brock 2009; Stockley and Bro-Jorgensen 2011). It is yet to be known whether early growth has differential effects on later fitness in males and females, in spite of the lack of sexual size dimorphism.

There is great heterogeneity among studies investigating the fitness consequences of early growth, with some considering mass at specific ages (e.g., Kruuk et al. 1999; Rödel and von Holst 2009) while others consider growth between two time periods (e.g., Lee et al. 2012). Considering both measures of growth and mass may be important as they can reflect different underlying processes. Growth provides a relatively instantaneous measure of the change in mass from one time point to the next, and may therefore be more reflective of the processes influencing development in that specific window. There is emerging evidence that early growth rates, independent of final body size attained, may influence later reproductive performance in some systems (e.g., Lee et al. 2012). Mass, on the other hand, is a more lagged measure and can be regarded as a memory statistic (i.e. state variable) that encompasses factors contributing to growth in previous time periods.

This study investigates sex differences in the link between early growth and later fitness in cooperative meerkats, using measures of growth and mass in early life. Meerkats live in groups of 3–50 individuals (Clutton-Brock et al. 2008) in which a dominant pair monopolises reproduction and helpers of both sexes assist in the rearing of dependent young. As reproductive skew is high in both sexes (Griffin et al. 2003), a primary driver of fitness is whether an individual becomes dominant or not in addition to its breeding success once dominant (Clutton-Brock et al. 2006; Hodge et al. 2008; Spong et al. 2008). Previous work has shown that current body mass, relative to immediate competitors, is an important predictor of dominance acquisition in females but not males (Clutton-Brock et al. 2006; Hodge et al. 2008; Spong et al. 2008). Two studies have investigated the influence of early growth on later fitness, showing that individuals who are heavier in early life are more likely to become dominant. The extent to which this varies between the sexes is not clear, however, as one study considered females only (Hodge et al. 2008) and the other considered a specific measure of early body mass (the amount of variance explained by helpers) on dominance acquisition in both sexes combined (Russell et al. 2007b). Moreover, it is not yet known whether early growth influences fitness beyond the acquisition of dominance status.

Here, we measured a suite of mass and growth traits during early development and several components of later fitness to investigate: (1) whether there are sex differences in development until sexual maturity; (2) the extent to which early growth influences the probability of attaining dominance, subsequent tenure and lifetime reproductive success; and (3) whether males and females differ in the relationship between early growth and measures of fitness.

## Materials and Methods

### Study site and species

This study was based on analysis of long-term data from a wild population of meerkats at the Kuruman River Reserve, South Africa (26°58′S, 21°49′E), collected between January 1998 and July 2011. Details on the study site and habitat are provided elsewhere (Russell et al. 2002). Individuals in the population were individually identifiable based on unique dye marks on their fur, habituated to close observation and weighed on a regular basis using laboratory scales (accuracy ±1 g). Observers visited groups about three times per week, noting life history events such as birth, deaths and emigrations. As such, the birth date of most individuals was known with an accuracy of 3 days.

### Variation in growth

We measured three parameters describing growth between birth and sexual maturity: mass at 1 month of age, growth between 1 month and 3 months and mass at 1 year of age. Our justification for selecting these three measures is as follows: (1) *Mass at 1 month:* Meerkat pups emerge from the burrow around 2–3 weeks of age and few measures of body mass are attained prior to this age. Until the age of about 1 month, pups rely almost exclusively on their mothers and allolactators for milk, and growth until this age therefore reflects maternal (and to some extent helper) investment (Russell et al. 2002, 2003). (2) *Growth between 1 and 3 months:* From about 1 month of age, pups leave the natal burrow to follow the foraging group, but until about 3 months of age, they are highly dependent on adult carers for food (Russell et al. 2002). Growth until independence at 3 months follows a different pattern to that after independence (English et al. 2012) and may reflect a sensitive period of early development. We measured growth until 3 months rather than mass at 3 months because we were specifically interested in the processes operating during this sensitive window and there is a longer delay for such processes to be reflected in mass rather than growth (see Introduction). (3) *Mass at 1 year:* We considered sexual maturity to be around 1 year of age, as few individuals successfully reproduced (9 out of 337 individuals) or attained dominance (3 out of 236 individuals) prior to this age. As there are seasonal and rain effects on growth at a daily scale (English et al. 2012), we used mass at the end of this pre-maturity growth period as an indication of the overall growth throughout the period. We estimated growth and mass measures for 882 individuals in total (448 males, 434 females) from individuals born into 308 litters produced by 99 mothers.

### Relationship between growth and later fitness

We investigated the relationship between early growth and later fitness by considering the following measures: (1) *Probability of attaining dominance*, a binary value assigned for whether an individual attained dominance at any point in its life or not; (2) *Tenure on attaining dominance*, the number of months an individual retained its dominance status (for those individuals who became dominant); (3) *Lifetime reproductive success (LRS),* the number of pups surviving until independence (3 months of age).

To avoid having a biased data set, our models analysing dominance acquisition, tenure and fitness only used data for individuals born more than 1210 days before the end of the study period, as at least 75 per cent of all dominant individuals had attained dominance by this age and survival of subordinate individuals drops off sharply beyond this age. As we were not working on a closed population, individuals emigrating from the study population could have become dominant elsewhere. To avoid any bias due to unknown fates of dispersing individuals, we excluded any individuals suspected to have emigrated, owing to temporary disappearance from the group in the month prior to the date they were last seen, resulting in a final sample size of 390 individuals.

Parentage estimates to calculate LRS were based on a combination of field and genetic data for females (field estimates are accurate if only one female is pregnant in the group) and genetic data only for males. Further details on the parentage analyses are provided in Nielsen et al. (2012). LRS was analysed for those individuals whose entire reproductive career was known and who were dominant for at least 3 months, and, for males only, who were themselves genotyped and had lost dominance status by the end of the period when genetic data were available (*n* = 34 females, 34 males). We excluded data on males who only attained dominance in their natal group, which occasionally happens if no immigrant males are present to fill a vacant dominant position (Spong et al. 2008). These “natal dominant” males are typically closely related to the dominant female and are therefore highly unlikely to breed in their natal group (Spong et al. 2008). As such, they represent an atypical case of social dominance in contrast to typical immigrant dominant males.

### Statistical analysis

All analyses were conducted in the statistical environment R 2.14.0 (R Core Team 2013). We first analysed sex differences in early growth parameters by conducting generalised linear mixed models (GLMM) with Gaussian error structure in lme4 (Bates and Maechler 2010), with a fixed effect of sex and random effects of birth cohort (year of birth, from 1 July to 30 June of next year), litter and mother. We investigated correlations among the growth measures using a Pearson's correlation test. To analyse fitness consequences of the three measures of early growth, we fitted them as fixed effects in separate models for males and females in light of previous work demonstrating sex differences in variance in reproductive success and duration of tenure (Clutton-Brock et al. 2006). Probability of becoming dominant was modelled using binomial error structure with random effects of birth cohort, litter and mother. Subsequent analyses did not include any random terms, owing to little replication within birth cohorts, litters or mothers. Dominance tenure was modelled as a proportional hazards regression (censored for those individuals still dominant at the end of the study period). LRS was modelled as a Poisson distribution with an observation-level random effect to account for overdispersion (Maindonald and Braun 2010), including tenure as a covariate. To assess the significance of fixed effect predictors, we used likelihood ratio tests (LRT) to compare nested models that did or did not include the fixed effect (Crawley 2007). The LRT statistic (χ^2^) with its associated *P*-value is provided for each term compared to the minimal model which includes significant terms only.

## Results

### Variation in growth

**Figure 1:**
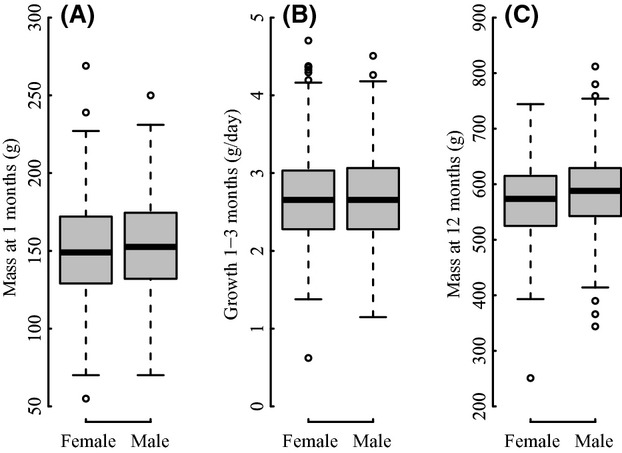
Box-and-whisker plots to demonstrate variability in the three growth parameters measured across 231 females and 159 males. While the sexes did not differ in mass at 1 month (A), or growth between 1 and 3 months (B), males had higher body mass at 1 year of age than females (C).

In line with previous work (Russell et al. 2002; MacLeod and Clutton-Brock 2013), we did not find any sex differences in mass at 1 month (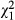 = 0.904, *P =* 0.342) or growth until independence (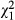
= 0.956, *P =* 0.328), but males were marginally heavier than females by 1 year of age (effect ± SE 15.50 ± 2.89; 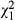
= 28.2, *P* < 0.001, Fig. 1). Mass at 1 month was negatively correlated with growth until independence (Pearson's *r*_880_ = −0.161), and positively correlated with mass at 1 year (*r*_880_ = 0.341); and growth until independence was positively correlated with mass at 1 year (*r*_880_ = 0.290). However, variance inflation factors for all measures were less than 1.4 suggesting that collinearity is unlikely to be an issue with their combined inclusion in subsequent models (Zuur et al. 2009).

### Relationship between growth and later fitness

#### Probability of becoming dominant

**Figure 2:**
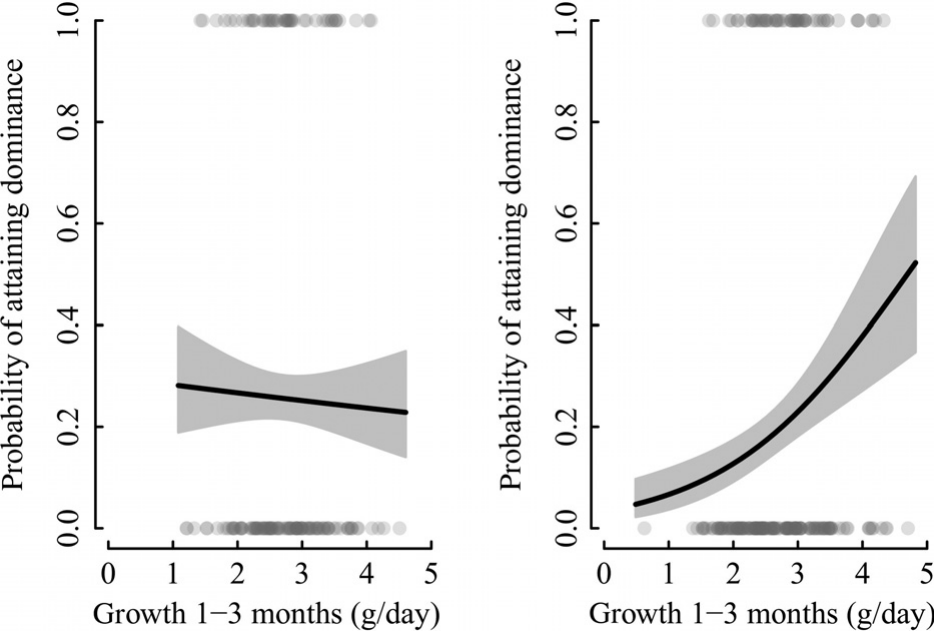
Relationship between growth until independence (g/day) and probability of dominance acquisition in (A) males and (B) females. Shown are the raw data (grey points) and the fitted effect (solid line) and standard error (grey shading) of growth until independence on dominance acquisition from a GLMM including this effect only. The effect of growth was significant in the model for females but not males.

Of the 231 females and 159 males which remained in the study population, 60 females and 48 males attained dominance. Females who exhibited higher growth until independence were more likely to attain dominance later in life (effect ± SE 0.97 ± 0.43; 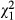 = 4.371, *P =* 0.037, Fig. 2), while there was no effect of either mass at emergence (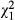 = 0.38, *P =* 0.561) or at maturity (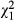 = 0.004, *P =* 0.947). In contrast, dominance acquisition in males was not influenced by mass at emergence (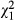 = 0.005, *P =* 0.944), growth until independence (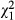 = 0.072, *P =* 0.788; Fig. 2) or mass at maturity (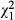 = 0.531, *P =* 0.466).

#### Dominance tenure

The duration of dominance tenure varied between 0 and 96 months among males and females (males, median: 12 months, IQR: 4–23 months; females, median: 14 months, IQR: 3–36 months). There were no significant effects of growth traits on the tenure of dominance in male or female meerkats (mass at emergence: males, 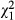 = 0.014, *P =* 0.906; females, 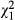 = 1.659, *P =* 0.198; growth until independence: males, 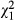 = 0.498, *P =* 0.481, females, 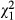 = 0.027, *P =* 0.869; mass at maturity: males, 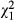 = 0.511, *P =* 0.475, females, 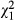 = 1.304, *P =* 0.254).

#### Lifetime reproductive success

Among dominant individuals who maintained their status for at least 3 months, LRS varied between 0 and 72 independent pups among females (*n* = 34), and between 0 and 31 independent pups among males (of those who were successfully genotyped, *n* = 31). The duration of dominance tenure had a significant, positive effect on LRS in males (effect ± SE 2.00 ± 0.47, 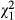 = 15.15, *P* < 0.001) and females (effect ± SE 2.14 ± 0.33, 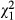 = 27.14, *P <* 0.001). In contrast, early development did not influence reproductive success among dominant male or female meerkats (mass at emergence: males, 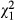 = 0.205, *P =* 0.651; females, 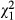 = 1.483, *P =* 0.223; growth until independence: males, 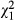 = 1.574, *P* = 0.210, females, 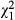 = 1.433, *P =* 0.231; mass at maturity: males, 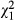 = 0.339, *P =* 0.561, females, 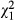 = 1.501, *P =* 0.221).

## Discussion

In this study, we found that early growth influenced dominance acquisition, a key route to fitness, in females but not males, in spite of both sexes exhibiting relatively monomorphic growth and males being slightly heavier at maturity. There were no effects of mass at emergence or maturity on dominance acquisition in either sex and none of the early growth measures had any subsequent influence on dominance tenure or breeding success once dominant. Our findings are in line with a previous study investigating the role of helpers on offspring fitness in meerkats (Russell et al. 2007b), which demonstrated that helper-mediated mass at independence was associated with the probability of breeding in males and females, and with the probability of attaining dominance in both sexes combined. By considering several measures of growth and mass and fitness measures beyond attaining dominance, our results present a more direct comparison of the link between early growth and later fitness between males and females. Below, we discuss these findings in light of burgeoning attention on the mechanisms of social competition in females.

This is one of the first studies, to our knowledge, to demonstrate a link between early growth and fitness-associated traits in a cooperative breeder, with growth having a stronger effect on fitness in the sex in which variance in reproductive success is higher as predicted based on patterns in polygynous species (Kruuk et al. 1999; Festa-Bianchet 2000; LeBlanc et al. 2001). Specifically, we found that the rate of growth during a key period, when pups are nutritionally dependent on adults, rather than mass at emergence or maturity, was important for later dominance acquisition in females. Rate of growth while pups are competing with one another over access to helpers (Hodge et al. 2007) may be a reliable proxy of relative competitive ability, and dominance hierarches among females may be partially established at this stage. Previous work has shown that mass relative to same-aged competitors, at the age when individuals compete for dominance, is an important predictor of dominance acquistion in females (Hodge et al. 2008). Our measure of absolute mass at maturity may not provide the resolution required to indicate relative competitive ability at the point of dominance acquisition (which may be several months or years later), for two reasons. First, slow growing and potentially less competitive individuals may exhibit catch-up growth after nutritional independence (Hector and Nakagawa 2012) and, second, absolute mass relative to the population mean may be a less sensitive measure of competitive ability than relative mass differences among competitors within a group (the measure used by Hodge et al. 2008). Relative competitive ability from an early age may be less important in males, who are less likely to inherit the dominant position in their natal group and may be under less intense competition with same-sex members of their cohort (Spong et al. 2008; Mares et al. 2012). Instead, other factors such as immediate condition while dispersing may be more important than competition with siblings for fitness prospects in males (Young et al. 2005; Bonte and De La Peña 2009).

The fact that growth until nutritional independence has fitness implications for female but not male meerkats (although other measures of mass did not have any effect) suggests two intriguing avenues for future research. First, we predict that selection on growth and later adult body size is stronger in females than males, in light of the link between growth and later reproductive success in females but not males (this study, Hodge et al. 2008; Spong et al. 2008). Second, if stronger selection leads to greater canalization of growth in females, we expect that sensitivity to environmental factors may be lower in females than males. Kruuk et al. (1999) found a similar effect in red deer, where birth weight (which is linked to lifetime reproductive success in male but not female red deer) is sensitive to population density and spring temperatures in females but not males.

Once individuals have attained the dominant breeding status, we found no further association between early growth and subsequent measures of reproductive success among dominant breeders in females. Previous studies have found that dominance tenure in females is influenced by the difference in body mass between the dominant female and her closest competitor at the onset of dominance (Clutton-Brock et al. 2006; Hodge et al. 2008). We did not find any effect of any early growth measures in females on tenure, however. Having acquired the dominant position, females employ low-level aggression to control the development and reproduction of their rivals (Kutsukake and Clutton-Brock 2005; Young et al. 2006), evicting them from the group before they become a threat. Given that physical fights are rare, absolute mass may not be an important predictor of success at maintaining dominance. Indeed, as most dominant females lose their status as a result of mortality (Hodge et al. 2008), typically caused by predation, there may be a highly unpredictable element to the length of time an individual maintains dominance status. Controlling for variation in tenure length, which is known to influence lifetime reproductive success (Hodge et al. 2008), we found no further effect of early growth measures on reproductive output after attaining dominance. In highly cooperative meerkats, helpers replace the effects of mothers on offspring growth and survival beyond emergence (Russell et al. 2002). Mothers adjust their investment in each reproductive attempt in light of such compensatory effects of helpers (Russell et al. 2003; Sharp et al. 2013), as in other species (Russell et al. 2007a, 2008). Measures of lifetime reproductive output may therefore be more sensitive to social factors rather than to attributes of maternal competitive ability.

As in females, we did not find any effect of early measures of growth or mass at maturity on reproductive success of males once they have acquired dominance status. Our results fit with previous work showing that tenure is not associated with adult body mass in males (Spong et al. 2008). This latter result is somewhat surprising: males more commonly lose dominance to foreign immigrants (Spong et al. 2008; Mares et al. 2012), yet our results imply that body mass does not accrue a competitive advantage to males. As males are more likely to disperse to become dominant (Spong et al. 2008; Mares et al. 2012), it is possible an inability to track individuals who have left the study population limits our conclusions on reproductive success in males. We attempted to minimise any sex bias in the effect of missing individuals in our analysis, however, by excluding those of both sexes who were thought to have emigrated.

We focused our analysis on dominance-associated fitness traits, as reproductive skew is high in meerkats, and the primary route to direct fitness is primarily through attaining the dominant position (Hodge et al. 2008; Spong et al. 2008). Nevertheless, subordinate individuals occasionally breed (Clutton-Brock et al. 1998, 2008; Young and Clutton-Brock 2006; Young et al. 2007) and it is as yet unknown whether early growth conditions and current body mass play a role in shaping fitness opportunities for subordinates even if they never become dominant, and whether there are sex differences in any effect.

To conclude, we have found sex differences in the fitness consequences of growth in a size-monomorphic species. Our results demonstrate how early divergence in growth rates may have lasting implications on fitness prospects, and that these depend on how the sexes differ in mechanisms and intensity of social competition. Finally, we emphasize the importance of considering several measures of mass and growth at different stages of development, which may provide complementary information on the relative competitive ability of individuals.
